# Controlled Growth of Two-Dimensional SnSe/SnS Core/Crown Heterostructures

**DOI:** 10.1021/acs.nanolett.4c03393

**Published:** 2024-10-16

**Authors:** Jennifer Schulz, Leonie Schindelhauer, Charlotte Ruhmlieb, Moritz Wehrmeister, Thomas Tsangas, Alf Mews

**Affiliations:** University of Hamburg, Institute of Physical Chemistry, Grindelallee 117, 20146 Hamburg, Germany

**Keywords:** tin sulfide, tin selenide, heterostructures, core/crown, nanosheets, 2D

## Abstract

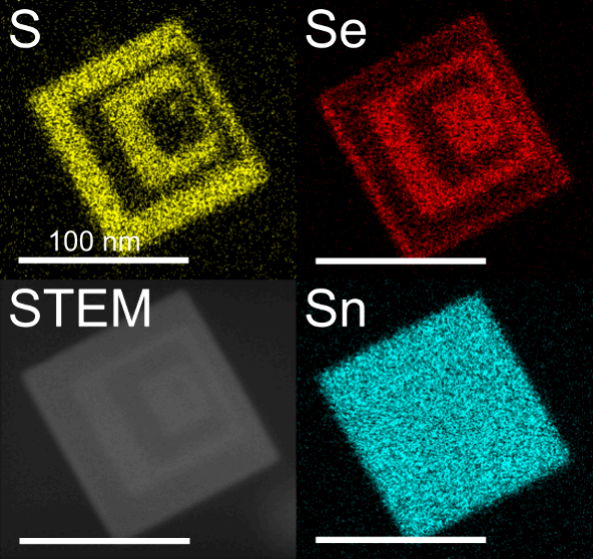

We present a novel, straightforward, and reproducible one-pot heating up technique to synthesize core/crown SnSe/SnS nanosheets by a careful adjustment of the sulfur and selenium precursor reactivity. Here, the SnSe nanosheets are prepared via a wet-chemical route using SnCl_2_ and selenium phosphines. By adding a highly reactive S-oleylamine complex during the growth of the SnSe sheets at 240 °C, SnS crowns were formed within the SnSe sheets. The number of SnS crowns can be tailored upon repeated addition of S-oleylamine. A combination of transmission electron microscopy, high-resolution transmission electron microscopy, atomic force microscopy, scanning transmission electron microscopy, and energy-dispersive X-ray spectroscopy proves the formation of highly crystalline core/crown structures.

Tin sulfide (SnS) and tin selenide (SnSe) are both known for low toxicity, natural abundance, and their optoelectronic capability. Especially the high absorption coefficient and high electrical conductivity make them two interesting representatives of 2D metal chalcogenides.^1−5^ Both SnS and SnSe are semiconducting materials with a direct (SnS: 1.30 eV; SnSe: 1.10 eV)^6,7^ and an indirect (SnS: 1.09 eV; SnSe: 0.90 eV)^6,7^ bandgap. Moreover, SnS and SnSe feature a relatively high electrical conductivity and a low thermal conductivity, making them excellent candidates for thermoelectric applications.^8^ Other applications include photocatalysis,^9^ electrodes for rechargeable batteries,^10−12^ and gas sensors.^13,14^ Regarding the crystallographic properties, SnS and SnSe, have compatible lattice parameters, which should allow for the epitaxial growth of a core/crown heterostructured nanosheet (HNS).^15^

Orthorhombic α-SnS and SnSe are promising semiconductors for solar light absorption due to their availability and band gap, which is well-positioned within the Shockley-Queisser limit for maximum solar cell efficiency.^5,16,17^ However, despite its potential, the efficiency of SnS-based solar cells remains below expectations, with reported efficiencies not exceeding 5%.^18^ This limitation can be attributed to factors such as the crystalline orientation of the material^19^ as well as crystal impurities, which cause recombination and limit performance.

One strategy to overcome these challenges is the development of SnS-based composites, e.g., SnS/CdS as it is reported in the literature.^20^ For SnS hybrid materials, it is crucial to maintain the basal plane of SnS as the primary absorber while ensuring that the secondary material, such as CdS, remains confined to the edges. This is important because charge carriers are preferentially transported toward these interfaces, which enhances charge separation and improves device performance.

The present study represents a significant step toward the fabrication of hybrid SnS nanostructures, demonstrating that a closely related material, SnSe, can be effectively used to create 2D core/crown nanosheets. This finding suggests that the strategies developed for SnSe-based nanostructures could be adapted to further improve the design and synthesis of SnS-based hybrid materials, which could potentially enhance the performance of future solar cell devices.

In this paper, we show that the reactivity of the anionic S and Se precursor plays a dominant role in the formation mechanism of 2D nanostructures. First, we present a synthesis method for 2D SnSe cores using Se-trioctylphosphine (Se-TOP) as precursor and compare it to the reactivities of several other anionic Se precursors. Then we demonstrate that the addition of a highly reactive S-oleylamine (S-OAm) precursor during the growth of SnSe nanosheets with a lower reactive Se-TOP precursor leads to the formation of HNSs where a SnS crown grows within the SnSe sheets. Finally, we show that even multiple-crown HNSs can be grown by the subsequent addition of S-OAm aliquots. High-resolution electron microscopy and elemental mapping was performed to receive a detailed structural insight into the crystal growth.

## Reactivity of the Precursors for the Formation of Tin Selenide and Tin Sulfide Nanosheets

The reactivity of the precursors is of major importance for both, nucleation and growth processes of nanostructures, and has been subject of several investigations.^21−24^ Especially the nucleation of anisotropic nanostructures such as 2D SnS nanosheets is a very complex process and goes far beyond classical nucleation theories. This has been shown in our previous paper where we used a hot injection technique and demonstrated that the initiation of the formation of SnS nanosheets could be monitored by a sudden color change of the reaction solution.^25^

**Figure 1:**
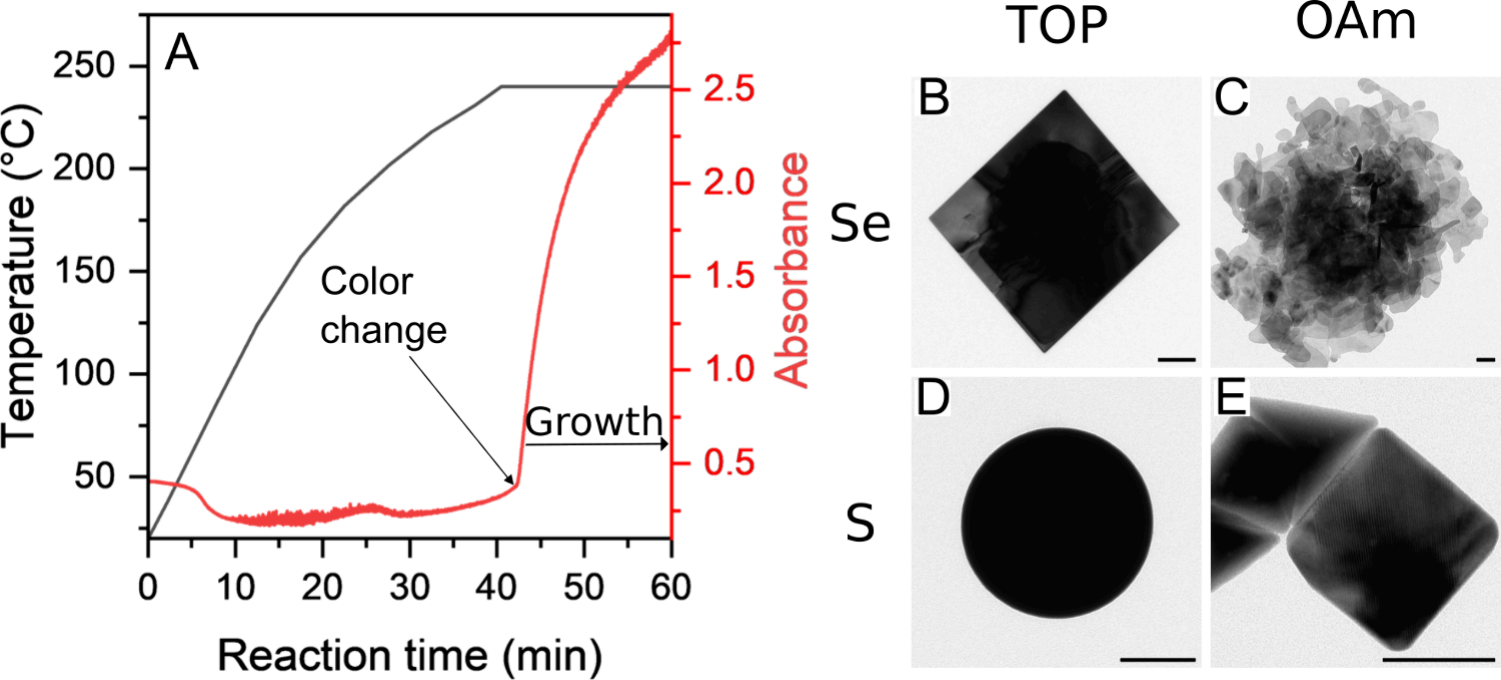
(A) Typical temperature and absorbance profile during the synthesis of SnSe nanostructures, using Se-TOP as precursor. The color change and the growth phase are marked. TEM images of the resulting nanostructures by using the following precursors: (B) Se-TOP, (C) Se-OAm, (D) S-TOP and (E) S-OAm (scale bars: 100 nm).

In this paper we used a one-pot heating up reaction strategy based on the method developed by Vaughn et al. (see the Supporting Information for experimental details).^26^ Basically, the Se or S precursors and SnCl_2_ are heated up in 20 mL oleylamine from room temperature up to 240 °C, and the color change at a certain temperature indicates the formation of nanosheets as shown in Figure 1A. For the experiments different anionic precursors such as X-TOP, X-OAm, X-ODE (1-octadecene), S-DDT (1-dodecanthiol), DDT, X-TBP (tributylphosphine), and (TMS)_2_-X (bis(trimethylsilyl), X: S, Se) were used.

Due to the different precursor reactivities, the color changes occur at different temperatures and times and also result in different final morphologies of the nanostructures. For example, when using Se-TOP as a precursor the color change was visible after 2:33 min at 240 °C, and results in almost quadratic SnSe nanosheets with an edge length of several 100 nm as shown in Figure 1B. By using Se-OAm the color change occurs already at 153 °C and the resulting nanostructures are relatively small and of irregular shape (see Figure 1C). In contrast, for the S-TOP precursor, the color change takes place after 4:50 min at 240 °C and the resulting nanostructures are regular tin spheres, as shown in Figure 1D. Finally, if the S-OAm precursor is used, the color change already occurs at 190 °C and the resulting SnS nanostructures are predominantly rectangular in shape.

Representative transmission electron microscopy (TEM) images of the structures resulting from using several other precursors are shown in Figure SI2 in the Supporting Information. It can be seen that only the use of Se-TBP, S-ODE, S-DDT, and DDT without any chalcogenide precursor resulted in rectangular nanosheets, similar to those produced with the S-OAm and Se-TOP precursors. Using S-TBP resulted in spherical tin particles comparable to the ones produced with the S-TOP precursor, while all the other chalcogenide precursor complexes led to structures with nonuniform morphology. This missing SnS formation indicates that S-TOP and S-TBP are barely reactive under the used reaction conditions. Also, the temperature and time at which the color change occurred is summarized in Table SI1 in the Supporting Information. Since a high precursor reactivity can be explained with a low reaction temperature and a short reaction time, the reactivity of the precursors decreases from (TMS)_2_ to DDT, OAm, ODE, TBP, and TOP, which is in agreement with reports in the literature.^21^ Additionally the Se precursor has a higher reactivity compared to the respective S precursor. Lower dissociation energies typically correlate with higher reactivity as the bond is easier to break during the reaction. The higher reactivity of the alkene and amine (ODE and OAm) precursors compared to the reactivity of the phosphine precursors (TOP and TBP) is due to the lower binding energies between ODE or OAm and the chalcogenides, respectively. The bond formation of alkenes and amines to S and Se takes longer or requires more energy,^21^ whereas tertiary phosphines quickly form bonds to the chalcogenide.^27^ This indicates a stronger binding energy and thus lower reactivity of X-TOP and X-TBP (X: S, Se), compared to the alkene and amine analogues. Another factor in precursor reactivity is steric hindrance. Larger ligands, such as trioctylphosphine, introduce steric hindrance, which can slow the reaction by making it harder for the precursor to interact with other reactants. This steric hindrance can reduce the overall reactivity of the precursor as it may prevent effective coordination and bond dissociation. In contrast, smaller ligands such as tributylphosphine offer less steric hindrance, allowing for easier access to the reactive site, which can enhance reactivity. The bond dissociation energies between the organic molecules and the chalcogenides decreases in the period from S to Se to Te.^27^ Selenium, being larger and less electronegative than sulfur, forms weaker bonds with metals, leading to lower bond dissociation energies for Se–C bonds compared to those for S–C bonds. This weaker bonding makes the selenium-containing precursors more reactive. Additionally, the larger atomic radius of selenium results in poorer orbital overlap with metals, which lowers the activation energy required for bond dissociation, thereby increasing the reactivity.

## Study of the Tin Selenide Nanosheet Formation

**Figure 2:**
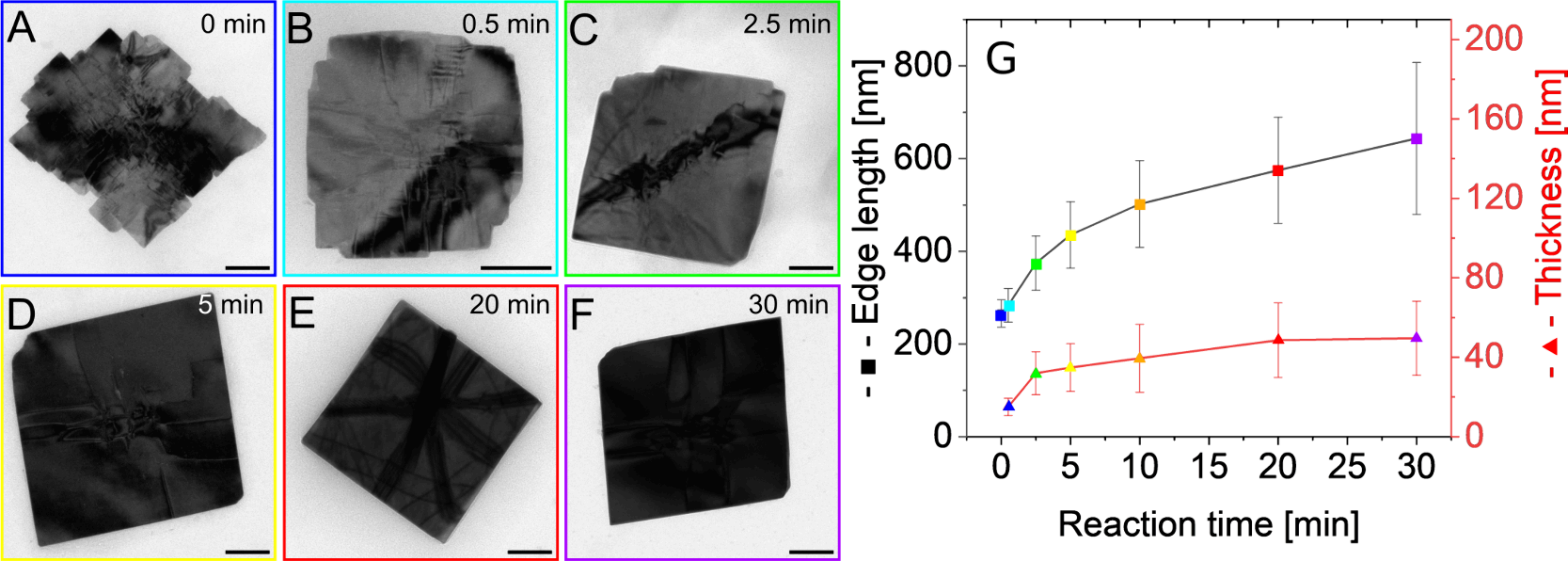
(A-F) Representative TEM images of SnSe nanosheets at different times during the synthesis after the color change (scale bars: 100 nm) and (G) edge length and thickness evolution during the reaction.

Since the use of Se-TOP as a precursor results in the most regular SnSe NSs, we first studied the formation process of those structures. For this, samples were taken at different times after the color change and examined with TEM and atomic force microscopy (AFM). Figures 2A-F show representative TEM images of the NSs at different times after the color change. Figure 2G depicts a statistic of the edge lengths and thicknesses of the NSs, as investigated by AFM.

Immediately after the color change, the NSs already have an average edge length of 265.9 ± 29.8 nm and an average thickness of 15.0 ± 4.4 nm, as measured via TEM from taken aliquots. At this point the edges of the NSs are irregular in shape, but get smooth after only 30 s reaction time featuring an average edge length of 283.4 ± 36.6 nm. The corresponding length distributions of the edges are shown in Figure SI3A-G in the Supporting Information. Since NSs are formed abruptly, as indicated by the rapid color change, the early growth of NSs is difficult to access. As shown in Figure 2G, the edge length of the NSs immediately after the color change has reached 44% of its final value and the thickness has already reached 30%. Both values increase steadily until the third minute, and then continue to increase at a slower rate until 30 min, with the edge length increasing more strongly than the thickness. After 30 min the sheets have a final average edge length of 643.8 ± 163.7 nm and an average thickness of 49.6 ± 18.7 nm. This study shows that the optimal time to begin crown growth is 5 min after the color change, when the edges are smooth enough to allow uniform crown growth (Figure 2D). Based on the calculated volumes it can be estimated, that only less than 30% of the precursor has reacted at this point.

## Reactivity of the Precursors for the Formation of SnSe/SnS Core/Crown Heterostructured Nanosheets

The reactivity of the different chalcogenide precursor complexes influences not only the formation of homogeneous SnS or SnSe NSs, but also the growth process of both materials on top of each other to form SnSe/SnS heterostructures. To experimentally evaluate the possibility of different combinations, various precursor combinations were examined to test the synthesis of SnSe/SnS or SnS/SnSe core/crown HNSs (see the Supporting Information for experimental details). From the TEM images in Figure SI4 in the Supporting Information, it can be seen that several precursor combinations with selenium phosphines as core precursor resulted in regular and rectangular NSs. The final SnSe/SnS HNSs were always larger than SnSe NSs synthesized without additional S precursors, indicating the growth of both materials on top of each other. A closer look reveals that especially the combination using Se-TOP for the core NSs and S-OAm for crown growth results in very thin and regular HNSs, where the core/crown structure is already visible in the TEM contrast. Interestingly, this is also the combination with the highest difference in reactivity for the rectangular HNSs (Table SI1 in the Supporting Information). In fact, DDT as a precursor resulted in an even higher reactivity, but the amount of the reactive species could hardly be determined. In the remaining part of the paper, we will concentrate only on the combination of Se-TOP as a precursor for the core NSs and S-OAm as crown precursor for the growth of heterostructures.

## Synthesis of SnSe/SnS Core/Crown Structures with Multiple Crowns

Since the S-OAm precursor is more reactive than the Se-TOP precursor, the growth of SnS should be preferred over the growth of SnSe if both anionic precursors, S-OAm and Se-TOP were present in the reaction solution. In fact, we could show that the formation of alternating SnSe and SnS crowns is possible, if sub stoichiometric amounts of S-OAm are added during the growth of SnSe using Se-TOP (see the Supporting Information for experimental details). In this case, the highly reactive S-OAm will grow a rectangular SnS crown and the remaining Se-TOP precursor will continue to grow a SnSe crown, when the S-OAm precursor is consumed.

**Figure 3:**
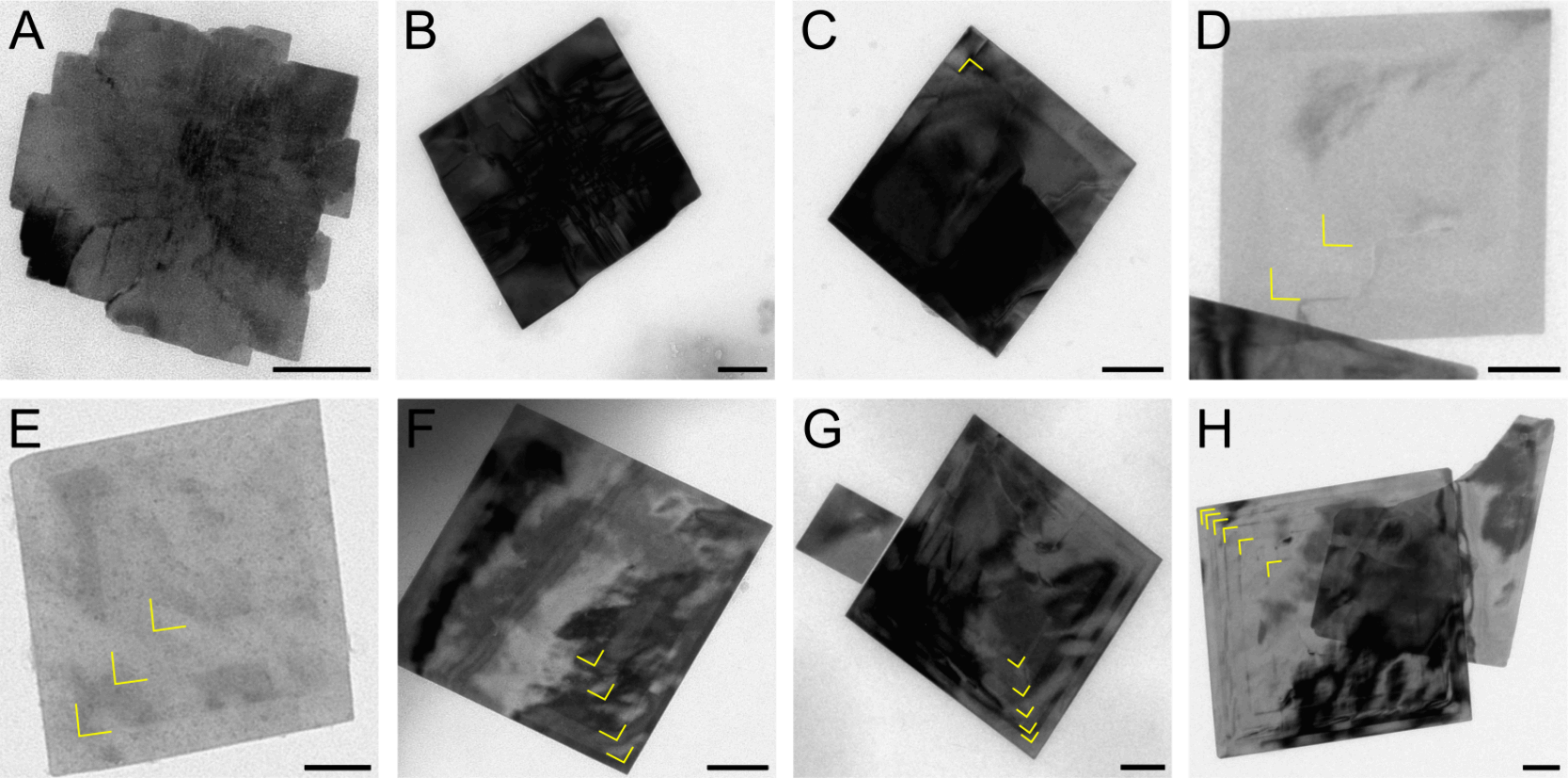
(A-H) TEM images of nanosheets taken during the growth of SnSe/SnS heterostructures at different times during the synthesis after the color change (scale bars: 100 nm). Yellow marks are inserted as a guide-to-the-eye to better see the phase boundaries.

This reaction can be monitored by taking aliquots during the reaction and investigating the nanostructures by TEM. Figure 3A shows a representative TEM image of a SnSe NS right after the color change, and Figure 3B shows a NS after a reaction time of 5 min right before the first addition of the S-OAm precursor. At this point 0.9 mL (0.0036 mmol) of the S-OAm precursor were added dropwise over the next 5 min to grow the first SnS crown (Figure 3C). Since Se-OAm and S-TOP do not lead to the formation of core/crown HNSs (Supporting Information Figure SI4K), S-OAm and Se-TOP seemingly do not undergo an exchange reaction but react as single entities. The dropwise addition is needed to avoid a too high concentration of S-OAm, otherwise side nucleation of SnS could occur. The following pictures (Figure 3E-H) show TEM images of the nanostructures during the growth of multiple alternating SnS and SnSe crowns. The size distributions extracted from TEM images of Figure 3 are comparable to the size distributions during the growth of SnSe NSs and are shown in Figure SI5 in the Supporting Information. Over 90% of these HNSs show core/crown structure. Due to the typical stacking of two-dimensional nanosheets, only a fraction of the structures can be precisely evaluated. Moreover, since the HNSs were purified by centrifugation, smaller NSs without a core/crown structure might have been washed away prior to the TEM measurements.

**Figure 4:**
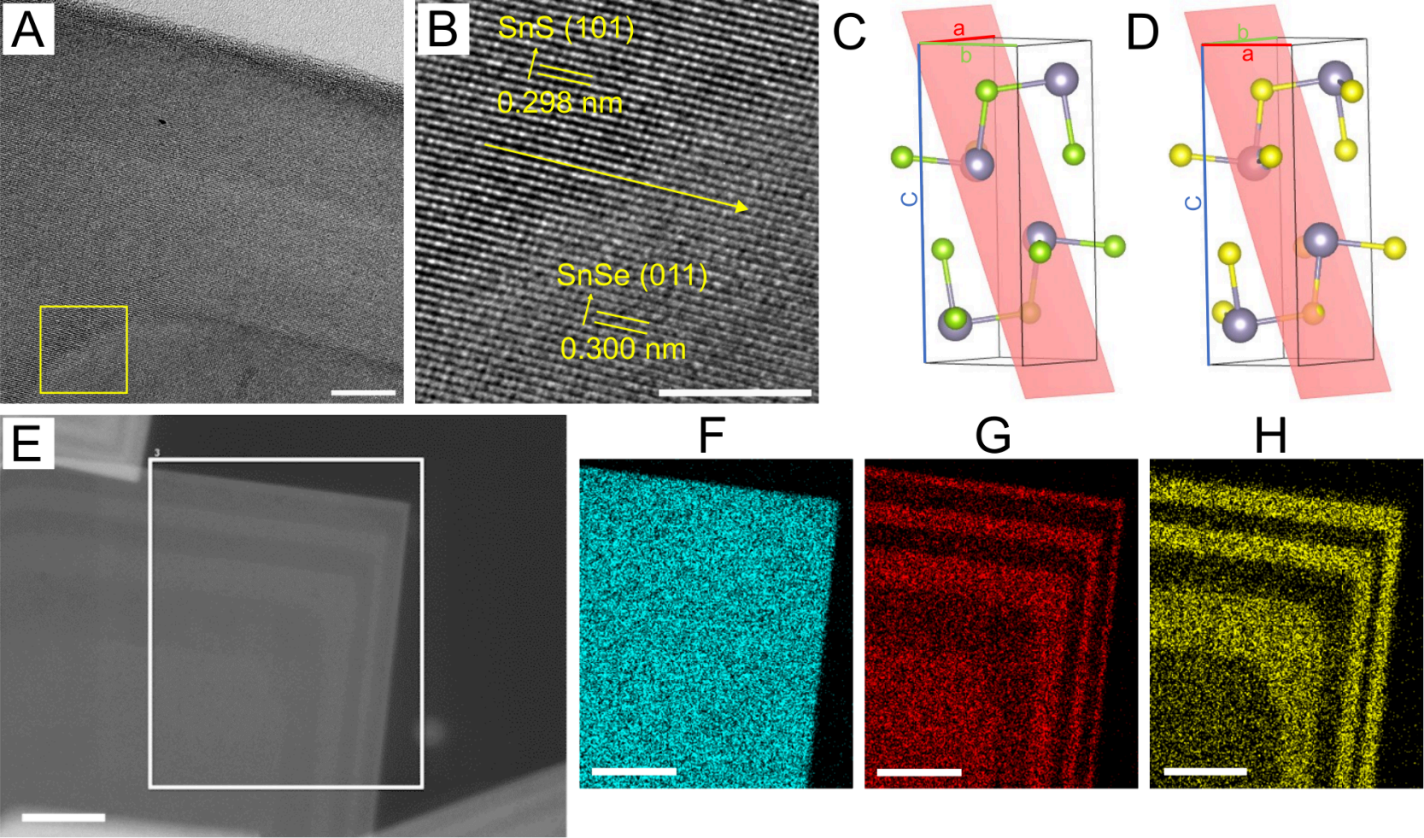
(A) HRTEM image of a SnSe/SnS heterostructured nanosheet (scale bar: 10 nm), (B) close-up of the marked area (scale bar: 5 nm), and the unit cells of (C) SnSe and (D) SnS with the lattice planes (011) of SnSe and (101) of SnS. Gray sphere: tin atom, green sphere: selenium atom, yellow sphere: sulfur atom. (E) Scanning transmission electron microscopy (STEM) image and corresponding EDX maps of (F) tin, (G) selenium, and (H) sulfur from a SnSe/SnS heterostructured nanosheet (scale bars: 100 nm).

While the different contrasts in Figure 3 are already a strong hint for the formation of SnSe/SnS core/crown NSs, we also recorded energy-dispersive X-ray spectroscopy (EDX) elemental maps to investigate the composition and high-resolution transmission electron microscopy (HRTEM) measurements to examine the crystallinity, as shown in Figure 4.

The spatially resolved EDX maps of tin (Figure 4F), selenium (Figure 4G), and sulfur (Figure 4H) from the SnSe/SnS HNSs (Figure 4E) clearly show that alternating SnS/SnSe crowns were grown by the procedure described above. Additional EDX maps are provided in Supporting Information Figure SI6–8.

The core/crown HNSs were additionally investigated by HRTEM (Figure 4A-B). Lattice spacings of 0.300 ± 0.048 and 0.298 ± 0.051 nm were measured, as marked in the close-up images. The determined lattice spacings coincide with the lattice spacings of the (011) lattice planes of SnSe (0.303 nm, Figure 4C) and the (101) lattice planes of SnS (0.293 nm, Figure 4D) (ICSD-PDF-No.: 00–048–1224 and 00–039–0354). In the SAED patterns (shown in Supporting Information SI9), an orthorhombic diffraction pattern is visible, consistent with the crystal structures of SnS and SnSe. Additionally, the diffraction spots appear “doubled”. This phenomenon arises from the slightly different unit cell parameters of SnS and SnSe, where the small variations in their lattice constants lead to a slight offset in the diffraction spots. These doubled spots are a direct consequence of the heterostructured nature of the nanosheets, confirming the coexistence of both SnS and SnSe phases within the core/crown architecture. Due to the large morphological anisotropy of the two-dimensional nanosheets, the powder X-ray diffraction data (see Supporting Information SI10) exhibit a strong texture effect, which does not allow for precisely distinguishing both phases. However, HRTEM in combination with the EDX maps made the SnS and SnSe areas visible. Since SnS and SnSe have very similar *d* spacings, the overall diffraction image must also be considered. Here, we see double reflection spots which display the very slight but visible difference in the *d* spacings of SnS and SnSe and confirm the presence of both materials. The HRTEM images also prove that SnS directly grows on SnSe without a significant lattice mismatch (Figure 4B). From the slightly different material contrasts an atomically sharp boundary between SnSe and SnS is visible in the HRTEM images.

Like the thickness of the SnSe NSs, the thickness of the HNSs also slightly increases with increasing lateral size. It appears that the lateral size increase dominates the morphology rather than any significant vertical growth. For example, the thickness of HNSs with one crown is 33 ± 3 nm, while the thickness of HNSs with three SnS crowns is 35 ± 8 nm, which remains within the standard deviation of the NSs and HNSs thicknesses measured with AFM. This suggests that the addition of SnS crowns does not lead to the formation of a shell around the SnSe core but rather contributes to lateral growth along the edges of the nanosheets.

**Figure 5:**
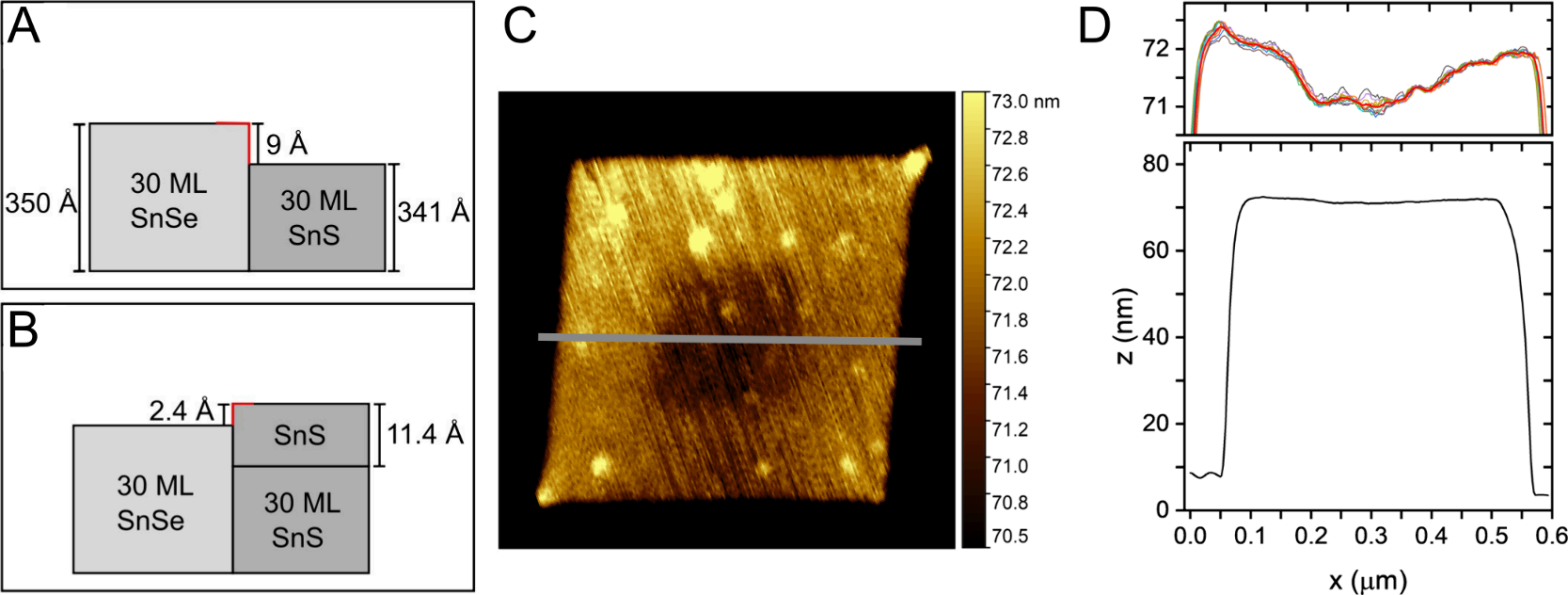
Scheme of the growth of the SnS crown: (A) Side view of a part of a SnSe/SnS core/crown heterostructured nanosheet with a SnSe core and a SnS crown with 30 monolayers (ML) each. The corner with increased surface energy, where the probability of growth is enhanced, is marked in red. (B) Growth of one ML of SnS at the SnS crown, starting at the corner with high surface energy, forming a new corner with high surface energy, facing toward the core. (C) AFM image of an SnSe/SnS core/crown heterostructured nanosheet with (D) corresponding height profile. The red graph represents the average value of the underlying.

Since the unit cell of SnS is about 0.3 Å smaller than the one of SnSe, and the SnSe cores are about 30 monolayers thick, an edge is formed with a height of about 9 Å (Figure 5A), which is not quite equal to the height of the unit cell of SnS (11.39 Å, ICSD-PDF-No.: 00–039–0354), again forming an edge of 2.4 Å in the direction of the SnSe core (Figure 5B). In this status, the core/crown nanosheets are present, as can be seen in the AFM image (Figure 5C) and the corresponding height profiles (Figure 5D).

UV/vis-NIR absorption spectroscopy was conducted by using an integrating sphere to analyze the optical properties of the nanosheets and the HNSs. The corresponding spectra in the Supporting Information (Figure SI11) show broad absorption across the visible and near-infrared regions, consistent with the optical properties of SnS and SnSe materials. From the Tauc plots (see Supporting Information SI11B-D), we identified the direct bandgap of SnSe to be 1.06 eV and of SnS to be 1.34 eV, which is in accordance to the literature.^6,7^ For the SnSe/SnS HNSs, the Tauc plot displays a bandgap of 1.10 eV. This is a reasonable value since it is between the bandgaps of both monomaterials, but slightly shifted to the lower bandgap of SnSe due to the higher content of SnSe in the HNS.

In summary, we have demonstrated the first wet-chemical synthetic procedure to form multiple-crown heterostructured SnSe/SnS nanosheets with lateral sizes of several hundred nanometers. The emerging large anisotropy is particularly interesting for e.g., controllable cation exchange and experiments for observing charge separation within a single HNS. The key to control the growth of crowns is the reactivity of the precursors. Reactivity tests based on the color change of the reaction solution at different reaction temperatures and times revealed that the chemical anionic precursor reactivity decreases from (TMS)_2_ to DDT, OAm, ODE, TBP, and TOP and from Se to S. This was further verified by tests to form core/crown structures with different combinations of the precursors. The synthesis of SnS crowns was optimized upon investigation of the growth of SnSe nanosheets to determine the start of the growth and TEM investigation to follow the NS formation. We found that the best moment for the SnS crown growth upon addition of S-OAm is at 5 min after the start of the SnSe growth as determined by the color change. Also, SnSe/SnS core/crown structures with multiple crowns were synthesized by subsequent additions of S-OAm. Elemental maps confirmed the distinct interface of the core material and the crown, which was further validated and investigated by HRTEM. Overall, our work demonstrates a new strategy to prepare heterostructures in a one pot synthesis by careful adjustment of the anionic precursor reactivity. This approach to achieve selected growth from different anionic precursors competing for the cationic precursor is not only restricted to the tin-chalcogenides system described in this work, but will certainly lead to several different heterostructures in the future.

## Supporting Information Available

Experimental Section; table of time and temperature of color change; figures of reaction scheme of synthesis, TEM images, size distributions, STEM images and EDX mappings, HRTEM images and SAED images, PXRD data, and UV–Vis absorbance spectra (PDF)

## Abbreviations

HNSs: heterostructured nanosheets
(TMS)_2_-S: bis(trimethylsilyl)sulfide
ODE: 1-octadecene
OAm: oleylamine
DDT: 1-dodecanthiol
TBP: tributylphosphine
(TMS)_2_-Se: bis(trimethylsilyl)selenide
TOP: trioctylphosphine
NSs: nanosheets
TEM: transmission electron microscopy
HRTEM: high-resolution transmission electron microscope
STEM: scanning transmission electron microscopy
EDX: energy-dispersive X-ray spectroscopy
AFM: atomic force microscopy.
