# Supplementary material for: Controlled Growth of Two-Dimensional SnSe/SnS Core/Crown Heterostructures
Source: Nano Lett. 2024 Oct 16;24(43):13624–30. doi: 10.1021/acs.nanolett.4c03393 (PMC11528548; doi:10.1021/acs.nanolett.4c03393)
Supplement: Supplementary file 1 — nl4c03393_si_001.pdf [file nl4c03393_si_001.pdf]

Supporting Information for

Controlled Growth of Two-Dimensional Core/Crown SnSe/SnS Heterostructures

Jennifer Schulz†, Leonie Schindelhauer†, Charlotte Ruhmlieb†, Moritz Wehrmeister†, Thomas Tsangas†, and Alf Mews†*

†University of Hamburg, Institute of Physical Chemistry, Grindelallee 117, 20146 Hamburg, Germany

** To whom correspondence should be addressed.*

E-Mail charlotte.ruhmlieb@uni-hamburg.de

EXPERIMENTAL SECTION

Materials. All chemicals were used without further purification. Bis(trimethylsilyl)sulfide ($(TMS)_2$-S, 100%), hexamethyldisilazane (HMDS, 99%), 1-octadecene (ODE, 90%), oleylamine (OAm, 70%) and sulfur powder (S, 99.99%) were purchased from Sigma Aldrich. Selenium powder (Se, 99.5%) was purchased from Thermo Fischer, 1-dodecanthiol (DDT, 100%), tetrachloroethylene (TCE, 100%) and tributylphosphine (TBP, 95%) from Acros Organic, tin(II)chloride ($SnCl_2$, 99.99%) from Alfa Aesar, bis(trimethylsilyl)selenide ($(TMS)_2$-Se, 100%) and toluene (99.85%) from Fisher Scientific and trioctylphosphine (TOP, 97%) from ABCR.

Synthesis of SnSe nanosheets (NSs). The SnSe NSs were prepared based on a method developed by Vaughn *et al.*[1], but with the following modifications. In a 50 mL three-neck round-bottom flask 10 mg (0.05 mmol) of $SnCl_2$ were dissolved in 20 mL OAm. The mixture was kept

[1] Vaughn, D. D.; In, S. Il; Schaak, R. E. A Precursor-Limited Nanoparticle Coalescence Pathway for Tuning the Thickness of Laterally-Uniform Colloidal Nanosheets: The Case of SnSe. ACS Nano 2011, 5 (11), 8852–8860. https://doi.org/10.1021/nn203009v.

under vacuum at room temperature for 1 h to remove traces of water and oxygen. Then 0.05 mL of Se precursor (previously prepared in a nitrogen-filled glovebox by dissolving 158 mg (2 mmol) of Se in 2 mL TOP) and 1 mL HMDS were added under nitrogen flow. Subsequently, the mixture was stirred for 15 min and slowly heated up to 240 °C at 10 °C/min. After the color change from light yellow to light grey at approximately 2-3 min at 240 °C the reaction solution was stirred for further 30 min at 240 °C. The reaction was stopped by rapidly cooling the mixture to room temperature upon removing the heating mantle and air cooling of the reaction vessel.

Synthesis of SnSe/SnS core/crown heterostructured nanosheets (HNSs). For the formation of SnSe/SnS HNSs, the SnSe cores were synthesized by performing the procedure as described above, but with half of the amount of Se and a reaction time of just 5 min after the color change. Then the SnS crown was synthesized by adding a certain amount of S precursor (previously prepared in a nitrogen-filled glovebox by dissolving 32 mg (1 mmol) of S in 1 mL OAm, stirring for 24 h and diluting 40 µL of it with 10 mL OAm) dropwise to the solution of growing SnSe NSs within 5 min at 240 °C using a syringe pump. The reaction was stopped 20 min after the addition of S precursor by rapidly cooling the mixture to room temperature.

Synthesis of SnSe/SnS core/crown HNS-structures with multiple crowns. For the synthesis of SnSe/SnS HNSs with multiple alternating crowns, the step of the growth of a SnS crown was repeated after 5 min (see Figure SI1 for the reaction scheme). The reaction was stopped 25-30 min after the first addition of S precursor by rapidly cooling the mixture to room temperature.

Purification. To purify the (heterostructured) nanosheets, 20 mL of toluene were added and the mixture was centrifuged at 11000 *g* for 10 min. Then the supernatant was carefully removed, and the precipitate was redispersed in toluene. The product was again separated by centrifugation and

decantation. The procedure was repeated three times. Finally, the (core/crown) nanosheets were stored in toluene.

Electron Microscopy. Transmission electron microscopy (TEM) images were obtained using a JEOL JEM 1011 at an acceleration voltage of 100 kV. High-resolution transmission electron microscopy (HRTEM) images were obtained using a double corrected (CESCOR and CETCOR, CEOS) JEOL JEM 2200FS. Scanning transmission electron microscopy (STEM) images and corresponding energy-dispersive X-ray spectroscopy (EDX) elemental maps were obtained using a JEOL JED-2300 analysis station. For the measurements, the samples were drop-casted on carbon-coated copper TEM grids with 400 grid squares. For the evaluation of the size distribution of the different nanostructures and for the determination of lattice spacings, the software ImageJ was used. At least 50 structures were counted for each sample on different TEM images from different areas of the TEM grids.

Atomic Force Microscopy. The thickness of the nanosheets was measured by atomic force microscopy (AFM) using the Bruker Nanowizard 4XP in QI mode with SCM-PIT-V2 tips. For the measurements, the dispersions were first drop-casted on glass substrates and ultrasonicated in isopropanol to remove larger clusters. Single nanosheets were then located using an Olympus BX51 optical microscope. For the post processing of the AFM images the software Gwyddion was used.

UV/Vis-NIR Spectroscopy. UV/Vis-NIR absorption spectra were recorded using a Cary 5000 spectrometer (Agilent) equipped with an external integrating sphere. The SnS, SnSe, and SnSe/SnS core/crown nanosheets were dispersed in tetrachloroethylene, respectively. Each dispersion was sonicated for 30 seconds prior to the measurement. Baseline correction was performed using a

quartz glass cuvette filled with pure tetrachloroethylene. The spectra were collected in the spectral range of 300 nm to 2000 nm.

ADDITIONAL TABLES

Table SI1. Time and temperature of the color change with different precursors.

**Precursor ligand**	**Color change**	
	**Se**	**S**
TOP	240 °C, 2:33 min	240 °C, 4:50 min
OAm	153 °C	190 °C
ODE	Not visible	205 °C
TBP	240 °C, 1 min	240 °C, 3 min
$(TMS)_2$	Room temperature	Room temperature
DDT	-	120 °C

ADDITIONAL FIGURES

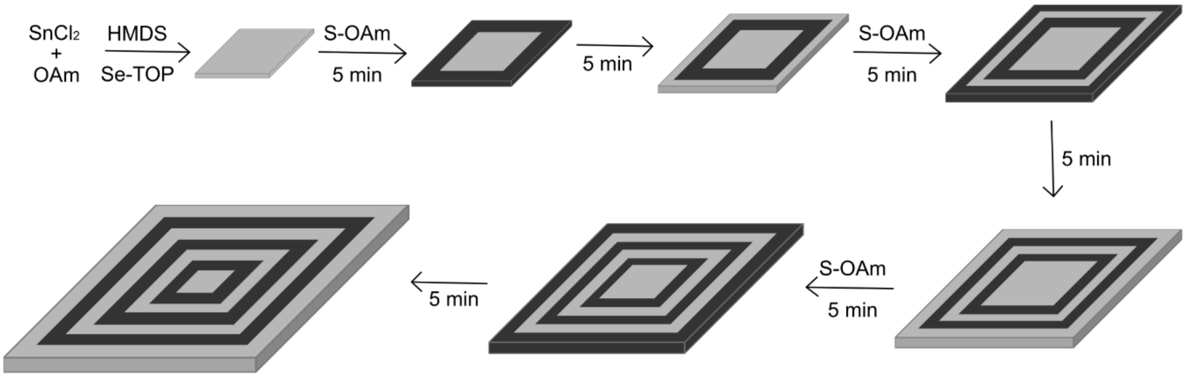

Figure SI1. Reaction Scheme of the synthesis of SnSe/SnS core/crown heterostructured nanosheets with alternating crowns.

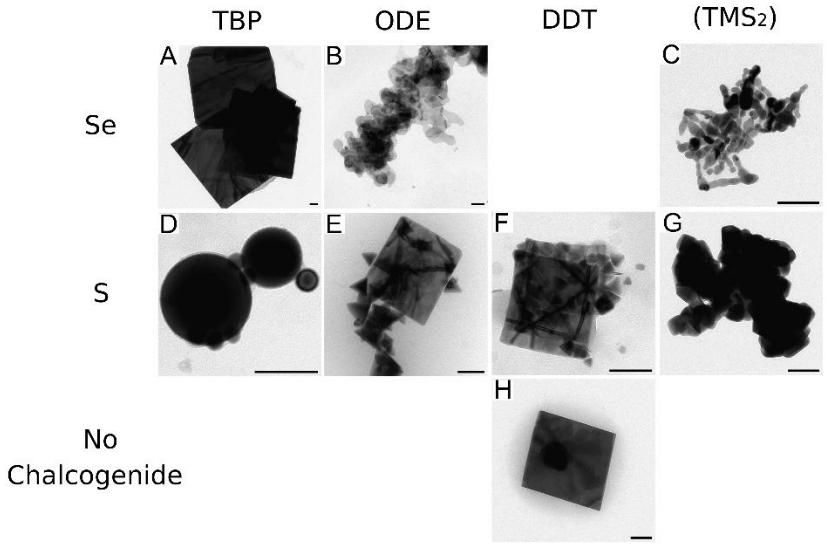

Figure SI2. TEM images of nanostructures synthesized according to the same protocol but with different precursors (A) Se-TBP, (B) Se-ODE, (C) $(TMS)_2$-Se, (D) S-TBP, (E) S-ODE, (F) S-DDT, (G) $(TMS)_2$-S and (H) DDT (scale bars: 100 nm).

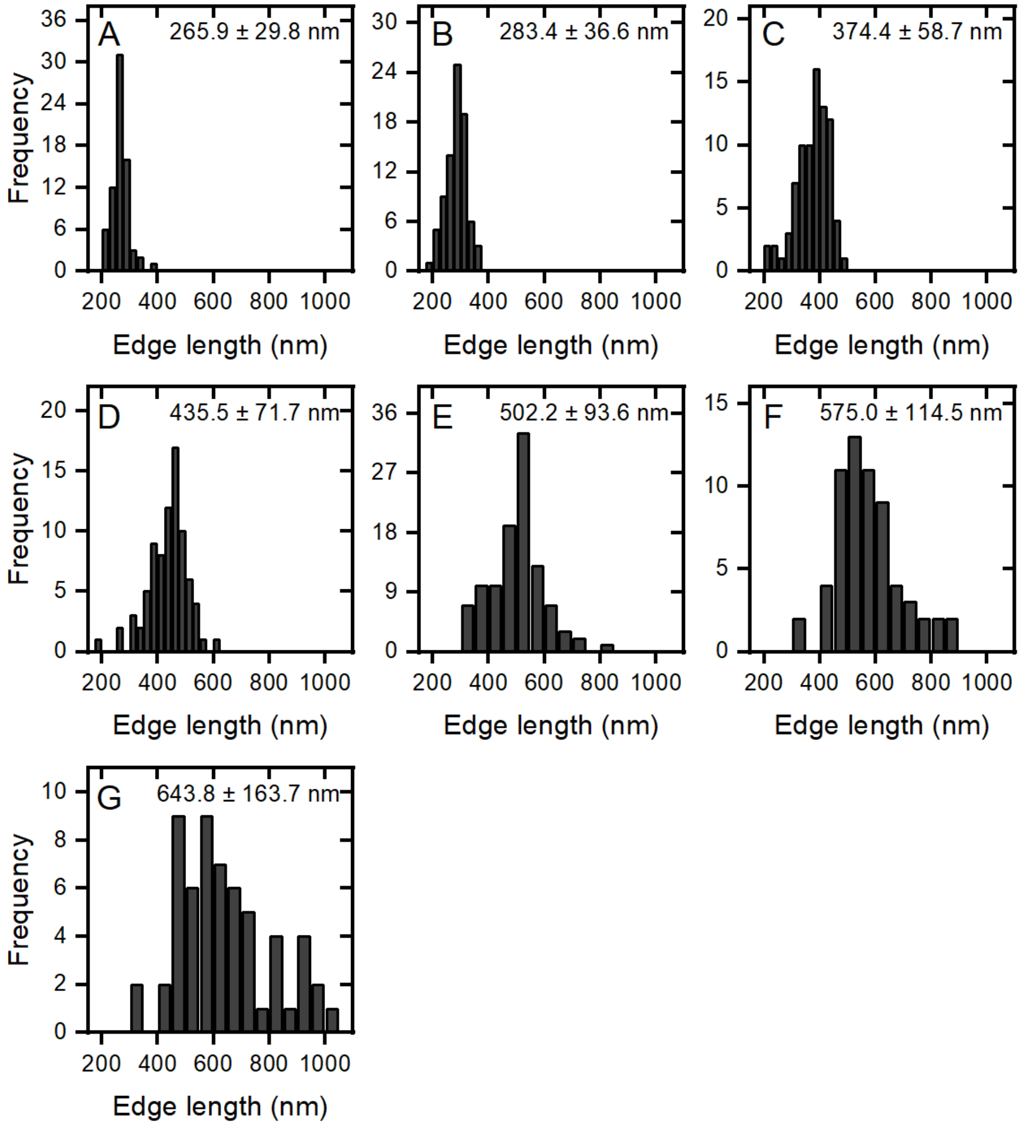

Figure SI3. Corresponding size distributions of the TEM images of the growth of SnSe nanosheets at different times during the synthesis after the color change (Figure 2).

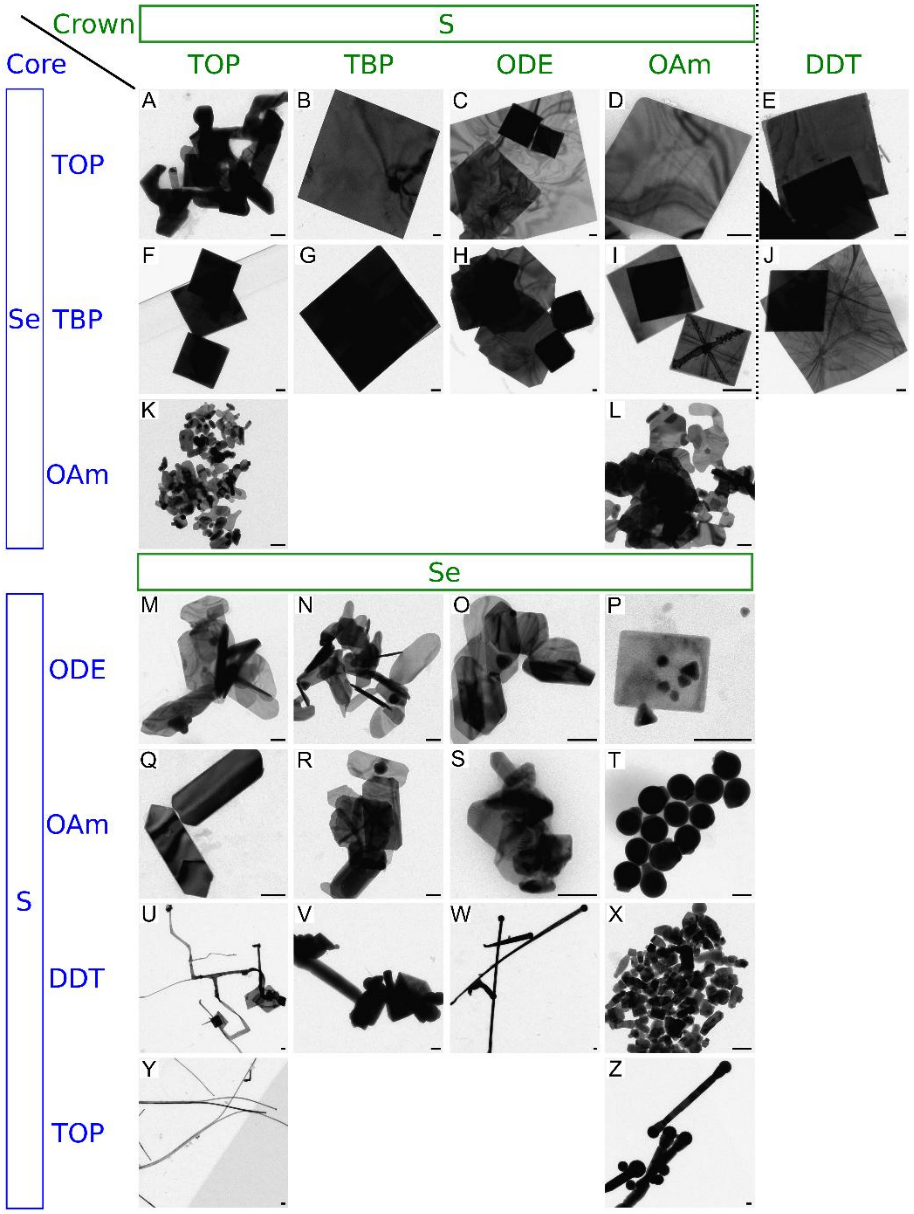

Figure SI4. TEM images of nanostructures according to the same protocol but with different combination of precursors: (A) Se-TOP – S-TOP, (B) Se-TOP – S-TBP, (C) Se-TOP – S-ODE, (D) Se-TOP – S-OAm, (E) Se-TOP – DDT, (F) Se-TBP – S-TOP, (G) Se-TBP – S-TBP, (H) Se-TBP – S-ODE, (I) Se-TBP – S-OAm, (J) Se-TBP – DDT, (K) Se-OAm – S-TOP, (L) Se-OAm – S-OAm, (M) S-ODE – Se-TOP, (N) S-ODE – Se-TBP, (O) S-ODE – Se-ODE, (P) S-ODE – Se-OAm, (Q) S-OAm – Se-TOP, (R) S-OAm – Se-TBP, (S) S-OAm – Se-ODE, (T) S-OAm – Se-OAm, (U) DDT – Se-TOP, (V) DDT – Se-TBP, (W) DDT – Se-ODE, (X) DDT – Se-OAm, (Y) S-TOP – Se-TOP and (Z) S-TOP – Se-OAm (scale bars: 100 nm).

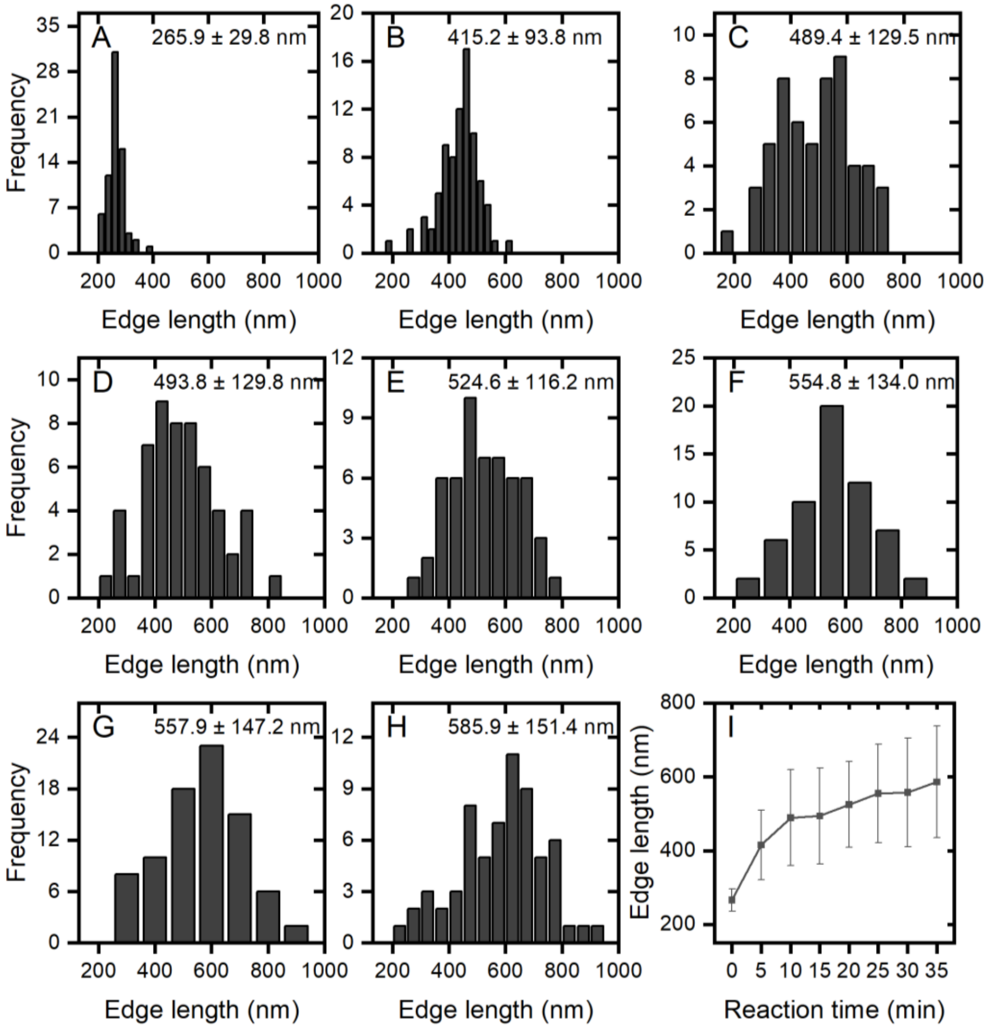

Figure SI5. (A-H) Corresponding size distributions of the TEM images of the growth of SnSe/SnS heterostructured nanosheets at different times during the synthesis after the color change (Figure 3) and (I) edge length evolution during the reaction.

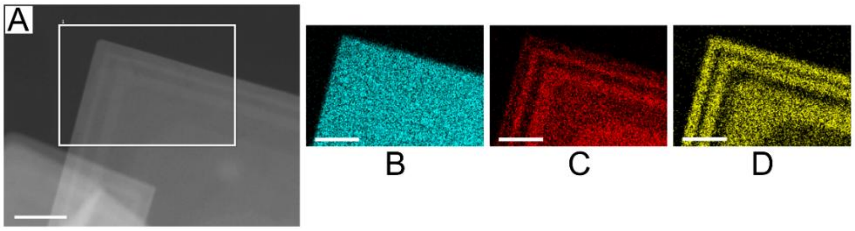

Figure SI6. (A) STEM image and corresponding EDX mappings of (B) tin, (C) selenium, and (D) sulfur from a SnSe/SnS heterostructured nanosheet (scale bars: 100 nm).

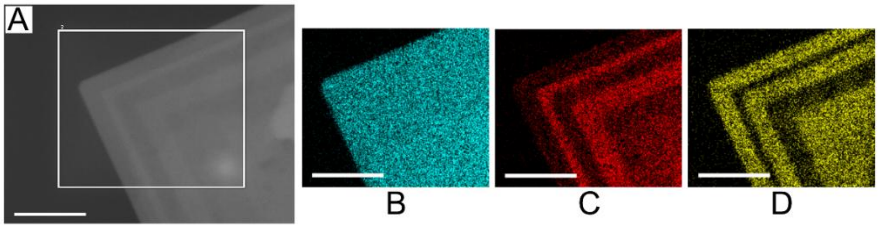

Figure SI7. (A) STEM image and corresponding EDX mappings of (B) tin, (C) selenium, and (D) sulfur from a SnSe/SnS heterostructured nanosheet (scale bars: 100 nm).

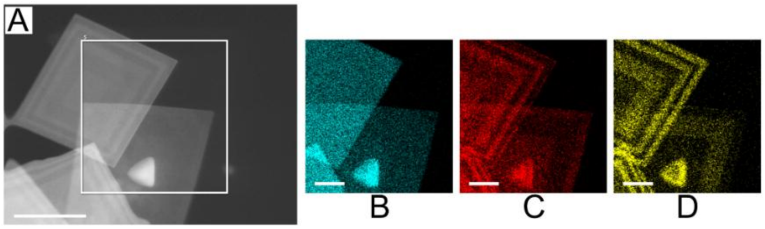

Figure SI8. (A) STEM image and corresponding EDX mappings of (B) tin, (C) selenium, and (D) sulfur from a SnSe/SnS heterostructured nanosheet (scale bars: 100 nm).

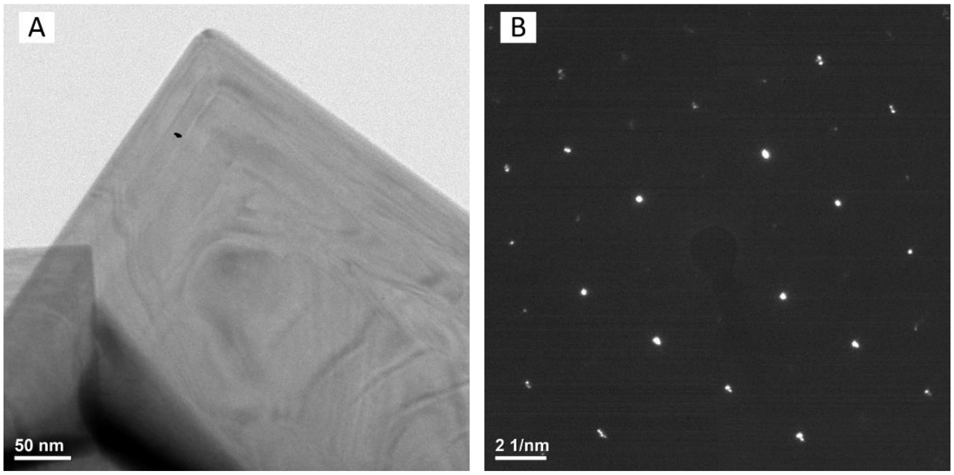

Figure SI9. (A) HRTEM image of a corner part of a SnSe/SnS core/crown HNS and (B) corresponding SAED image. The SAED confirms the crystallinity of the HNSs and their orthorhombic crystal structure. While showing the same general pattern, the diffraction spots of SnS and SnSe have a slightly different distance to the blanked primary beam, since the unit cell of SnSe is slightly larger than the unit cell of SnS.

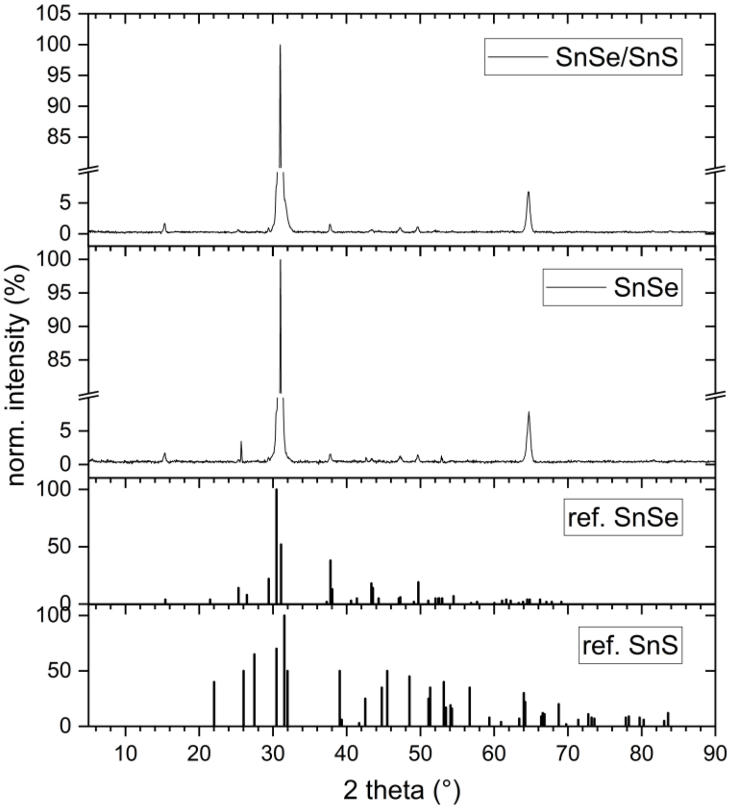

Figure SI10. PXRD data of the SnSe nanosheets and HNSs in comparison to reference crystal data for orthorhombic SnSe (PDF 00-048-1224) and SnS (PDF 00-039-0354). Due to the large anisotropy, a strong texture effect in the intensities of some reflections is observed.

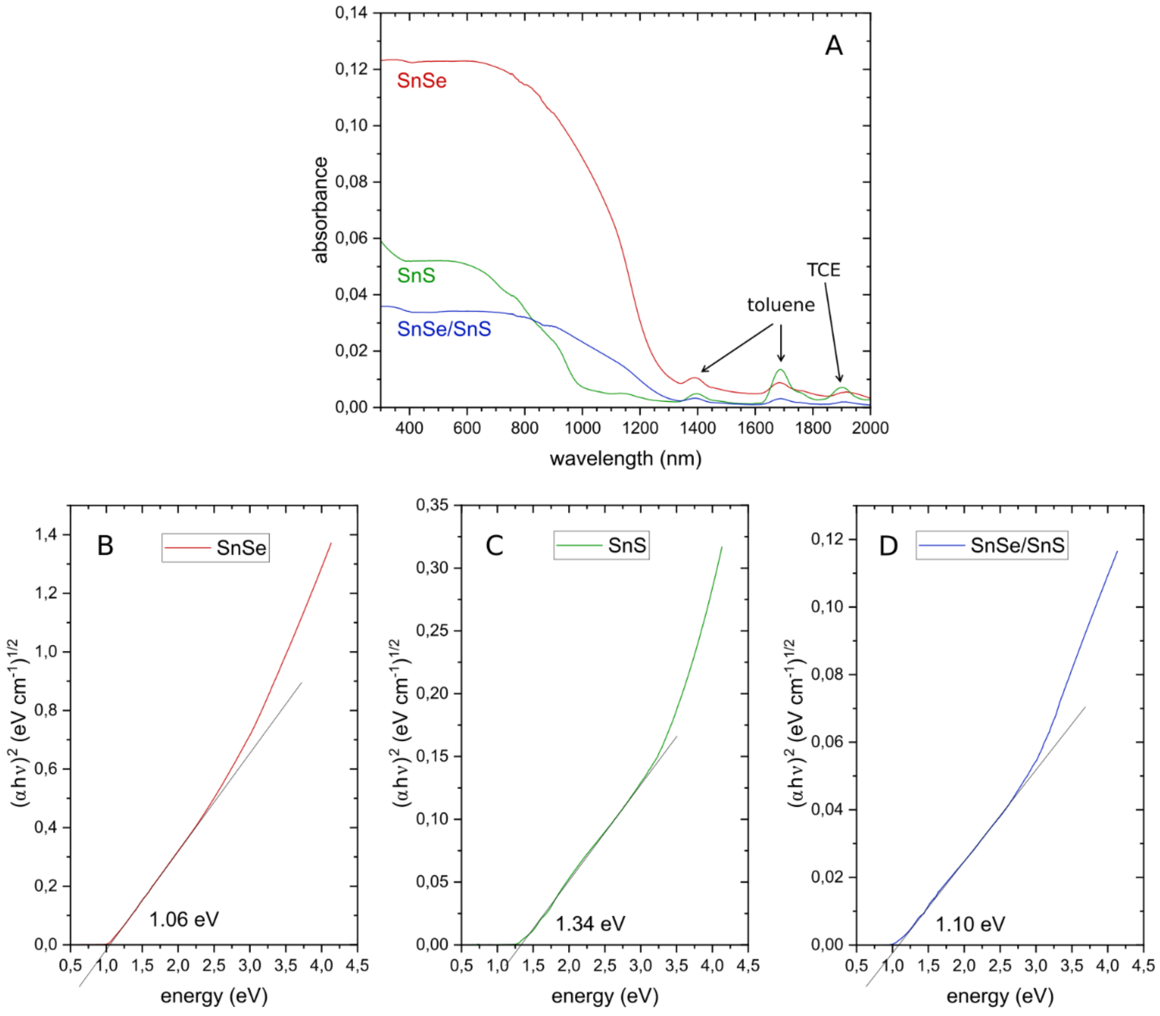

Figure SI11. (A) UV/Vis absorbance spectra of SnS nanosheets, SnSe nanosheets, SnSe/SnS core/crown HNSs measured in tetrachloroethylene (TCE) in the range of 300 to 2000 nm. Peaks at around 1400, 1700 and 1900 nm originate from toluene residues and TCE. (B-D) Tauc plots of SnSe, SnS and SnSe/SnS nanosheets dispersed in TCE with identified bandgaps of 1.06 eV for SnSe and 1.34 eV for SnS. The SnSe/SnS HNSs feature a bandgap of 1.10 eV which represents an average value of both bandgaps.
